# A Synoptical Guide to the Study of Obstetrics

**Published:** 1884-03

**Authors:** 


*A Synoptical Guide to the Study of Obstetrics.* By ROBERT BARNES, M.D. Pp. 122. London: SMITH, ELDER & Co. 1884.

These notes are evidently the work of a scientific man thoroughly conversant with his subject. Though the work is little more than the skeleton notes which a lecturer would use, and so would be specially favoured by the student attending lectures or preparing for examination, yet their scope is so wide and their arrangement and classification so scientific and methodic that the practitioner wishing to test his knowledge or refresh his memory would find it most useful. To those who are fond of writing small books on great subjects we would commend this one of Dr. Barnes as a pattern to copy.

